# 

**Published:** 1928

**Authors:** 


### The Bristol Medico-Chirurgical Journal

A JOURNAL OF THE MEDICAL SCIENCES FOR THE
WEST OF ENGLAND AND SOUTH WALES.

PUBLISHED UNDER THE AUSPICES OF
*THE BRISTOL MEDICO-CHIRURGICAL SOCIETY.*

EDITOR:
J. A. NIXON, C.M.G., M.D., F.R.C.P.

WITH WHOM ARE ASSOCIATED
E. W. HEY GROVES, M.S., F.R.C.S., B.Sc.
E. WATSON-WILLIAMS, M.C., Ch.M., F.R.C.S.E.
A. E. ILES, O.B.E., M.B., B.S. Lond., F.R.C.S.
CAREY F. COOMBS, M.D., F.R.C.P., Assistant Editor.

EDITORIAL SECRETARY:
A. L. FLEMMING, M.B., B.Ch.

---

"Scire est nescire, nisi id me

Scire alius scierit."

---

*VOL. XLV.*

BRISTOL: J. W. ARROWSMITH LTD.
London: J. W. Arrowsmith (London) Ltd.
1928.

R31
.B8

### Contents of Vol. XLV.

Page

Some Developments in Dermatology during the last Thirty Years. By W. Kenneth Wills, O.B.E., M.B., B.Ch. .. .. .. .. 1

The Long Fox Memorial Lecture: The Reality of Delusions. By Henry Devine, O.B.E., M.D., B.S., F.R.C.P. .. .. .. .. .. 19

Problems in the Treatment of Hæmoptysis. By Hugh H. Carleton, M.A., M.D., M.R.C.P. .. .. .. .. .. .. .. 39

Recent Experiences of Intussusception. By Hubert Chitty, M.S., F.R.C.S. 51

The Comparative Value of Radiation Therapy in Dermatology. By Ernest Dore, M.D., F.R.C.P. .. .. .. .. .. .. .. 85

The Evidences of Heart Failure, and the Signs and Symptoms of Three Cases personally examined. By P. Christine Vine .. .. .. 97

Ocular Manifestations following Encephalitis Lethargica. By A. Michael Critchley, M.B., Ch.B. .. .. .. .. .. .. .. 113

A Case of Mitral Stenosis with Auricular Fibrillation, treated with Digitalis by the Cary Eggleston Method. By K. H. Pridie .. .. .. 125

Sugar Tolerance Tests. By Hugh D. Pyke .. .. .. .. 129

A Note on the Pathology of Erythema Nodosum. By A. C. Fisher .. 137

Indications for Manipulative Surgery. By A. G. Timbrell Fisher, M.C., M.B., B.Ch., F.R.C.S. .. .. .. .. .. .. .. 165

The Colloidal State of Matter. By F. Francis, D.Sc. .. .. .. 177

Redundant Colon: A Group of Cases Exhibiting Symptoms Traceable to this Condition. By Gilbert B. Bush, B.A., M.B., Ch.B., D.M.R.E. Cantab. .. .. .. .. .. .. .. .. .. 181

The Lymphocytosis of Infection. By T. Wilson-Smith, M.D., M.R.C.P. 187

The Value of the Chloride Content of the Cerebro-Spinal Fluid in Diagnosis and Prognosis. By Herbert Rogers, M.B., Ch.B. .. .. .. 197

Meningococcal Septicæmia with a Report of a Case. By C. Bruce Perry, M.B., M.R.C.P. .. .. .. .. .. .. .. .. 207

The Long Fox Memorial Lecture: The Relationship of Erythema Nodosum to Tuberculosis. By J. Odery Symes, M.D. .. .. .. 233

The Early Diagnosis and Treatment of Insanity. By J. V. Blachford, M.D. 253

Peri-Arterial Sympathectomy. By E. W. Hey Groves, M.S., F.R.C.S. .. 273

The Symptoms and Signs of Duodenal Ulcer. By T. Carwardine, M.S.. . 279

The Medical Treatment of Duodenal Ulcer. By R. C. Clarke, M.B., M.R.C.P. .. .. .. .. .. .. .. .. .. 289

Treatment of Infantile Paralysis of the Lower Limb. By M. Forrester Brown, M.S., M.D. Lond. .. .. .. .. .. .. .. 295

Chemotherapeutic Researches on Cancer—A Review .. .. .. 217

Reviews of Books .. .. .. .. .. .. .. 59, 143, 219, 309

Editorial Notes .. .. .. .. .. .. .. .. 68, 151, 225, 319

Obituaries .. .. .. .. .. .. .. .. .. 155, 321

Meetings of Societies .. .. .. .. .. .. .. 71, 158, 228, 322

The Medical Library of the University of Bristol .. 75, 159, 230, 324

Local Medical Notes .. .. .. .. .. .. .. 76, 162, 231, 326

### The Medical Library of the University of Bristol,

WITH WHICH IS INCORPORATED

**The Library of the Bristol Medico-Chirurgical Society.**

---

*The following donations have been received since the publication of the List in December,* 1927.

January, 1928.

| | |
|---|---|
| Dr. T. S. Cullen (1) .. .. .. .. | 1 volume |
| Professor E. W. Hey Groves (2) .. .. | 6 volumes |
| Michell Clarke Memorial Fund (3) .. .. | 20 ,, |
| Miss Rudge (4) .. .. .. .. .. | 1 volume |

Unbound periodicals have also been received from Colonel P. Bush, Dr. Carey Coombs, Dr. C. Corfield, Professor E. W. Hey Groves, Dr. F. S. Hunter and Miss H. B. Murgoci.

### THE ONE HUNDRED AND TWENTY-THIRD LIST OF BOOKS.

The figures in round brackets refer to the figures after the names of the donors. The books to which no such figures are attached have either been bought from the Library Fund or received through the *Journal*.

| | | | |
|---|---|---|---|
| **Bainbridge, F. A., and Menzies, J. A.** *Essentials of Physiology.* 5th Ed. | | (3) | 1925 |
| **Bauer, K. H.** .. .. | *Frakturen und Luxationen* .. .. .. .. .. | (2) | 1927 |
| **Brend, W. A.** .. | *Handbook of Medical Jurisprudence.* 5th Ed. | (3) | 1924 |
| **Clark, A. J.** .. .. | *Comparative Physiology of the Heart* .. .. | (3) | 1927 |
| **Cockayne, O.** .. .. | *Leechdoms, Wortcunning and Starcraft.* Vol. 1. | (4) | 1864 |
| **Cullen, T. S.** .. .. | *Early Medicine in Maryland* .. .. .. .. .. | (1) | 1927 |
| **East, W. N.** .. .. | *Forensic Psychiatry* .. .. .. .. .. .. .. | (3) | 1927 |
| **Eden, T. W., and Holland, E.** *Manual of Midwifery* .. 6th Ed. | | (3) | 1925 |
| **Finlayson, J.** .. .. | *Clinical Manual* .. .. .. .. .. 4th Ed. | (3) | 1926 |
| **Fuchs, J.** .. .. .. | *Technische Operationen in der Orthopädie* .. | (2) | 1927 |
| **Gibson, A. G., and Collier, W. T.** *Methods of Clinical Diagnosis* .. | | (2) | 1927 |
| **Gwathmey, J. T.** .. | *Anæsthesia* .. .. .. .. .. .. .. 2nd Ed. | (3) | 1925 |
| **Harrison, L. W.** .. | *Venereal Diseases* .. .. .. .. .. 3rd Ed. | (3) | 1926 |
| **Henderson, D. K., and Gillespie, R. D.** *Textbook of Psychiatry* .. | | (3) | 1927 |
| **Henry, A. K.** .. .. | *Exposures of Long Bones* .. .. .. .. .. .. | | 1927 |
| **Holt, L. E., and Howland, J.** *Diseases of Infancy and Childhood.* 9th Ed. | | (3) | 1926 |
| **Hutchison, R.** .. .. | *Food and the Principles of Dietetics* .. 6th Ed. | (3) | 1927 |
| **Ker, C. B.** .. .. .. .. | *Manual of Fevers* .. .. .. .. .. 3rd Ed. | (3) | 1927 |
| **Kleinschmidt, O.** .. | *Chirurgische Operationslehre* .. .. .. .. .. | (2) | 1927 |
| **Léri, A.** .. .. .. .. | *Études sur les Affections des Os* .. .. .. .. | (2) | 1927 |
| **Leveuf, J., et Girode, Ch.** *Le Traitement des Fractures du Col du Femur* | | (2) | 1927 |
| **Osler, W., and McCrae, T.** *Modern Medicine.* 5 Vols. .. .. 3rd Ed. | | (3) | 1925-7 |
| *Queen Charlotte's Practice of Obstetrics, The* .. .. .. .. .. .. .. .. | | (3) | 1927 |

| | | | |
|---|---|---|---|
| **Rawling, L. B.** | *Landmarks and Surface Markings of the Human Body* .. .. .. .. .. .. .. 6th Ed. | (3) | — |
| **Romanis, W. H. C., and Mitchiner, P. H.** | *The Science and Practice of Surgery.* Vol. II. | (3) | 1927 |
| **Rowlands, R. P., and Turner, P.** | *The Operations of Surgery.* Vol. II. 7th Ed. | (3) | 1927 |
| **Short, A. R.** | *New Physiology* .. .. .. .. .. .. 5th Ed. | (3) | 1922 |
| **Stern, B. J.** | *Social Factors in Medical Progress* .. .. .. | | 1927 |
| **Stitt, E. R.** | *Practical Bacteriology and Blood Work and Animal Parasitology* .. .. .. .. .. .. 8th Ed. | (3) | 1927 |
| **Thomson, J.** | *Clinical Study and Treatment of Sick Children.* 4th Ed. | (3) | 1925 |
| **Williams, G.** | *Minor Surgery and Bandaging.* 19th Ed. | (2) | 1927 |

TRANSACTIONS, REPORTS, JOURNALS, ETC.

| | | |
|---|---|---|
| Boletin del Instituto Clinica Quirurgica. Ano III. (1927. Nos. 21-25) | (2) | 1927 |
| British Journal of Surgery, January, 1928 .. .. .. .. .. .. .. .. .. | | 1928 |
| Churchill's Medical Directory .. .. .. .. .. .. .. .. .. .. .. .. .. | | 1928 |
| Methods and Problems of Medical Education. 8th Series .. .. .. | | 1927 |
| Progressive Medicine. Vol. 4. December, 1927 .. .. .. .. .. .. .. .. | | 1927 |
| Smithsonian Institution—Annual Report, 1926 .. .. .. .. .. .. .. .. | | 1927 |
| Surgical Clinics of North America. Vol. 7, Nos. 5 and 6.. .. .. .. .. | | 1927 |
| Transactions of the Association of American Physicians. Vol. 42 .. .. | | 1927 |

### Local Medical Notes.

We deeply regret to announce the death of Mr. J. P. I. Harty, which occurred on March 5th, 1928. An Obituary Notice will appear in our next issue.

---

Lt.-Col. F. P. Mackie, O.B.E., M.D., M.Sc., D.P.H. (Bristol), F.R.C.S., F.R.C.P., I.M.S., Director of the Haffkine Institute, Bombay, has been appointed an Honorary Surgeon on the personal staff of His Excellency Lord Irwin, Viceroy and Governor-General of India.

EXAMINATION RESULTS.

**Conjoint Board.**

M.R.C.S., L.R.C.P.—T. L. Cleave, G. Steinberg.

L.D.S., R.C.S.—*First Professional Examination. Part I.* (*Dental Mechanics*): F. G. Lilley, M. E. Minton. *Part II.* (*Dental Metallurgy*): H. Horwood. *Part IIIa* (*Anatomy and Physiology*): I. M. Ayliffe, W. R. R. Mewton. *Part IIIb* (*Dental Anatomy and Physiology*): T. A. A. Eaves, W. Forsyth, W. F. G. Harvey, J. J. Hinton, H. J. Phillips.

### Bristol Medico-Chirurgical Society.

### President.

W. K. WILLS, *O.B.E.*, M.A., M.B., B.Ch. Cantab.

### President-Elect.

H. LAWRENCE ORMEROD, M.D., B.Ch., R.U.I.

### Committee.

*Ex-Officio* { J. A. NIXON, *C.M.G.*, B.A., M.D.Cantab. (Editor of *Journal*).

W. A. SMITH, M.A., M.B Oxon. (Hon. Librarian).

*Elected.*

A. L. FLEMMING, M.B., Ch.B. Brist. }
F. LACE, F.R.C.S. } Ex-Presidents.
T. CARWARDINE, M.S. Lond. }
D. C. RAYNER, F.R.C S.
C. FERRIER WALTERS, F.R.C.S.
E. W. HEY GROVES, M.D., M.S. Lond.
W. VINCENT WOOD, *M.C.*, B.A. Cantab.
DUNCAN WOOD, F.R.C.S.
R. S. S. STATHAM, *O.B.E.*, M.D., M.Ch. Brist.

### Honorary Secretary.

W. A. JACKMAN, M.B., F.R.C.S. Ed.

### Honorary Treasurer.

A. E. ILES, *O.B.E.*, M.B., B.S., F.R.C.S.

### University Library Sub-Committee.

W. A. SMITH, M.B., M.A. Oxon. G. PARKER, M.D., M.A. Cantab.

A. R. SHORT, M.D.

Medical Practitioners wishing to join the Society are requested to communicate with the Hon. Secretary, Mr. W. A. Jackman, 15 Mortimer Road, Clifton. The Annual Subscription is £1 11s. 6d.. payable to Hon. Treasurer.

---

*Extracts from Journal By-laws.*

4.—The *Journal* shall be delivered free to Subscribers and also to Members of the Society whose subscriptions are not in arrear.

6.—The Annual Subscription to the *Journal* is 10/6, payable to Hon. Treasurer.

---

Communications intended for insertion in the *Journal* should be sent to the Editor, Dr. J. A. Nixon, 7 Lansdown Place, Clifton, Bristol.

Books for Review and Exchange Journals should be sent to the Assistant-Editor, *Bristol Medico-Chirurgical Journal*, Medical Library, The University, Bristol.

Communications referring to the delivery of the *Journal* should be sent to the Editorial Secretary, A. L. Flemming, 48 Pembroke Road, Clifton, Bristol.

Cases for Binding Vols. I—XLIV are now ready, and may be obtained, price 1/9 each (postage extra), upon application to J. W. Arrowsmith Ltd., Quay Street, Bristol; or the *Journal* will be bound, including Case, for 7/- per volume.

### Bristol Medico-Chirurgical Society.

### PAST PRESIDENTS.

1874—75. FREDERICK BRITTAN, M.D., B.A. Dub.
1875—76. ROBERT WILLIAM COE.
1876—77. SAMUEL MARTYN, M.D. Ed.
1877—78. AUGUSTIN PRICHARD, M.D. Berlin.
1878—79. HENRY EDWARD FRIPP, M.D. Würzburg.
1879—80. WILLIAM MICHELL CLARKE.
1880—81. JOSEPH GRIFFITHS SWAYNE, M.D. Lond.
1881—82. EDWARD LONG FOX, M.D. Oxon.
1882—83. JAMES GEORGE DAVEY, M.D. St. And.
1883—84. WILLIAM JOHNSTONE FYFFE, M.D., B.A. Dub.
1884—85. GEORGE FORSTER BURDER, M.D. Aberd.
1885—86. WILLIAM HENRY SPENCER, M.D., M.A. Cantab.
1886—87. FRANCIS POOLE LANSDOWN.
1887—88. LEMUEL MATTHEWS GRIFFITHS.
1888—89. ROBERT SHINGLETON SMITH, M.D., B.Sc. Lond.
1889—90. NELSON CONGREVE DOBSON, Ch.M. Brist.
1890—91. SAMUEL HENRY SWAYNE.
1891—92. FRANCIS RICHARDSON CROSS, M.B. Lond., LL.D. Brist.
1892—93. EDWARD MARKHAM SKERRITT, M.D., B.S., B.A. Lond.
1893—94. JAMES GREIG SMITH, M.B., C.M., M.A. Aberd., F.R.S.E.
1894—95. ALFRED JAMES HARRISON, M.B. Lond.
1895—96. ARTHUR WILLIAM PRICHARD.
1896—97. ALFRED EDWARD AUST LAWRENCE, M.D., C.M. Aberd.
1897—98. JOHN EDWARD SHAW, M.B., C.M. Ed.
1898—99. ROBERT ROXBURGH, M.B. Ed.
1899—00. WILLIAM HENRY HARSANT.
1900—01. DAVID SAMUEL DAVIES, M.D. Lond.
1901—02. BARCLAY JOSIAH BARON, M.B., C.M. Ed.
1902—03. GEORGE MUNRO SMITH, M.D. Brist.
1903—04. JAMES PAUL BUSH, C.M.G., C.B.E., Ch.M. Brist.
1904—05. REGINALD EAGER, M.D. Lond.
1905—06. JOHN DACRE.
1906—07. JAMES TAYLOR.
1907—08. HENRY WALDO, M.D., C.M. Aberd.
1908—09. JOHN MICHELL CLARKE, M.D., M.A. Cantab.
1909—10. JAMES SWAIN, C.B., C.B.E., M.D., M.S. Lond.
1910—11. BERTRAM MITFORD HERON ROGERS, M.D., B.Ch., B.A. Oxon.
1911—12. CHARLES ALEXANDER MORTON.
1912—13. WALTER CARLESS SWAYNE, M.D., B.S. Lond. and Brist.
1913—14. PATRICK WATSON-WILLIAMS, M.D. Lond.
1914—15. WILLIAM HARRY CHRISTOPHER NEWNHAM, M.A. M.B. Cantab.
1915—16 GEORGE PARKER, M.A., M.D. Cantab.
1916—17 FRANCIS HENRY EDGEWORTH, M.A., M.D. Cantab., D.Sc. Lond.
1917—18 FREDERICK LACE.
1918—19 ROBERT GUTHRIE POOLE LANSDOWN, M.D., B.S. Durh.
1919—20 I. WALKER HALL, M.D., Ch.B. Vict.
1920—21 LEOPOLD ERNEST VALENTINE EVERY-CLAYTON, M.D. Lond.
1921—22 CYRIL HUTCHINSON WALKER, M.B., B.A. Cantab.
1922—23 JOHN ALEXANDER NIXON, C.M.G., B.A., M.D. Cantab.
1923—24 JOHN LACY FIRTH, M.D., M.S. Lond.
1924—25 JOHN ODERY SYMES, M.D. Lond.
1925—26 THOMAS CARWARDINE, M.S. Lond.
1926—27 ARTHUR LAUNCELOT FLEMMING, M.B., Ch.B. Brist.

1928.

### LIST OF SUBSCRIBERS

TO THE

### Bristol Medico-Chirurgical Journal.

### HONORARY MEMBERS OF THE SOCIETY.

Swain, James, *C.B.*, *C.B.E.*, M.D., M.S. ... ... ... ... Wyndham House, Easton-in-Gordano.
Watson-Williams, P., M.D. .. 2 Rodney Place, Clifton.

### SUBSCRIBERS.

*Those marked * are Members of the Society.*

*Adams, A. W., M.S.. Lond. ... ... ... ... 9 Mortimer Road, Clifton.
Adye, W. J. H. ... ... ... ... ... ... ... St. Margaret's, Bradford-on-Avon.
*Alexander, A., M.A.... ... ... ... ... ... 23 Manilla Road, Clifton.
*Alexander, D. A., M.B., Ch.B. Vict. ... ... 112 Pembroke Road, Clifton, Bristol.
Alexander, G. L., B.A., Cantab. ... ... ... Principal Medical Officer, Freetown, Sierra Leone.
*Alford, A. F., M.B., Ch.B. Bristol ... ... "Drcfla," Kellaway Avenne, Westbury Park, Bristol.
Alford, H. J., M.D. ... ... .. ... ... ... 4 Park Terrace, Taunton.
*Askins, R. A., M.D., B Ch., B.A.O, Dub.... Guildhall, Bristol.
*Aubrey, T., M.B. Lond. ... ... ... ... ... Bitton, near Bristol.

*Baker, Lily A., M.D., B.Ch., B.A.O., R.U.I.... 21 Victoria Square, Clifton.
*Barber, M. C., M.B., Ch.B. Brist. ... ... ... Ingledene, High Street, Staple Hill Bristol.
*Barker, G. H., M.D., B.Sc. Lond. ... ... ... 124 Redland Road, Bristol.
*Bartholomew, E. U. ... ... ... ... ... ... 12 Lansdown Place, Clifton.
Beatson, D. H., M.B., Ch.B. ... ... ... ... 8 Richmond Park Road, Clifton.
*Benson, Elizabeth, M.B., Ch.B. Brist. ... 8 Theresa Place, Bristol Road, Gloucester.
*Bergin, F. G. ... ... ... ... ... ... ... ... 31 Oakfield Road, Clifton.
Bernard, C. ... ... ... ... ... ... .. ... 564 Fishponds Road, Fishponds, Bristol.
Berry, F. C., M.D., B.Ch., B.A. Dub. ... ... Locarno, Burnham, Somerset.
*Birrell, J. A., M.D., M.S. Lond. ... ... 10 Clifton Park, Clifton, Bristol.
*Blachford, J. V., *C.B.E.*, M.D. Durh. ... ... 1 Victoria Square, Clifton.
Blackett, J. F., M.D., B.S. Lond. ... ... ... Hamsterley, Bloomfield Park, Bath.
*Blagg, Arthur F., M.D. Durh.... ... ... ... 6 Upper Belgrave Road, Clifton.
*Blathwayt, A. de V.... ... ... ... ... ... 6 Brock Street, Bath.
Bletchly, G. Playne, M.B. Lond. ... ... ... Hazelwood, Nailsworth, Glos.
*Bodman, A. P., M.B., Ch.B. ... ... ... ... 11 Hurle Crescent, Clifton, Bristol.
*Bodman, C. O., M.D. Durh. ... .. ... ... 17 Upper Belgrave Road, Clifton.
*Bodman, F. H., M.B., Ch.B. Brist. ... ... ... 170 Cheltenham Road, Bristol.
*Bodman, J. Hervey, M.D. Lond. ... ... ... 11 Hurle Crescent, Clitton, Bristol.

| | |
|---|---|
| *Bowker, G. E., M.D. Ed. ... ... ... ... ... | 11 North Parade, Bath. |
| *Bradbeer, E. G., M.B., Ch.B. Brist. ... ... | 32 Linden Road, Westbury Park, Bristol. |
| *Bradley, W. H., B.M., B.Ch. Oxon. ... ... | St. Wulstan's, Stratton-on-Fosse, Bath. |
| *Branthwaite, C.B., M.D., D.P.H. ... ... | Stoke Park, Stapleton. |
| *Bray, G., Lt.-Col., *D.S.O.* ... ... ... ... | 26 Beaconsfield Road, Clifton. |
| *Brentnall, T. C. ... ... ... ... ... ... ... | Bellfield, Brightlingsea, Essex. |
| *Britton, R. B., M.B., B.S. Lond. ... ... ... | Troy House, Kingswood, Bristol. |
| *Buckmaster, G. A., M.A., M.D. Oxon. ... ... | University of Bristol. |
| *Burdon-Cooper, J., M.D., B.Sc. Durh. ... | 12 The Circus, Bath. |
| *Burges, R. ... ... ... ... ... ... ... ... | Druid's Mead, Stoke Bishop, Bristol. |
| *Burnett, R. H., M.B., B.S. Durh. ... ... ... | 479 Bath Road, Brislington, Bristol. |
| *Bush, G. B., M.B., Ch.B. Brist. ... ... ... | 5 Lansdown Place, Clifton. |
| | |
| *Cairns, F. J. ... ... ... ... ... ... ... ... | Devon House, Whitehall Road, Bristol. |
| *Carey, R. S., *O.B.E.*, B.A. Cantab. ... ... | 502 Bath Road, Brislington, Bristol. |
| *Carleton, H. H., M.A., M.D. B.Ch. Oxon. | 1 Lansdown Road, Clifton. |
| *Carling, A., M.A., M.B., B.C. Cantab. ... ... | 12 Great George Street, Bristol. |
| *Carter, T. M. *O.B.E.*, M.D. ... ... ... | 19 Westfield Park, Redland, Bristol. |
| *Carwardine, Thomas, M.B., M.S. Lond. ... | 16 Victoria Square, Clifton. |
| *Cave, Edward J., M.D. Lond. ... ... ... ... | 16 The Circus, Bath. |
| *Chambers, E. R. ... ... ... ... ... ... ... | 9 Clifton Park, Clifton. |
| *Charles, J. R., M.A., M.D., B.C. Cantab. | Deepholm, Clifton Park, Clifton. |
| *Chitty, H., M.S. Lond. ... ... ... ... ... | 46 Pembroke Road, Clifton. |
| *Christofferson, P. E., M.B., Ch.B. Brist. ... | 8 Lansdown Place, Clifton. |
| *Claremont, L. E. ... ... ... ... ... ... ... | 5 Rodney Place, Clifton. |
| *Clarke, R. C., *O.B.E.*, M.B., Ch.B. Brist. ... | 29 Victoria Square, Clifton. |
| *Coates, Vincent M., *M.C.*, M.A., M.D., B.C. Cantab. ... ... ... ... ... ... | 10 The Circus, Bath. |
| Connellan, P. S. ... ... ... ... ... ... ... | 12 Bloomsbury Street, London, W.1. |
| *Cookson, R. G., L.R.C.P., L.R.C.S. Ireland | Clifton Dispensary, Bristol. |
| *Coombs, Carey, M.D. Lond. ... ... ... ... | 3 Pembroke Road, Clifton. |
| *Cooper, William Russell ... ... ... ... | 13 Westfield Park, Redland, Bristol. |
| *Corfield, C., M.D. Durh. ... ... ... ... | 5 Kensington Place, Clifton. |
| Cridland, A. B. ... ... ... ... ... ... ... | Salisbury House, Chapel Ash, Wolverhampton. |
| *Cross, F. R., M.B. Lond., LL.D. Brist. ... | Worcester House, Clifton. |
| *Crossman, F, W., M.B. Durh. ... ... ... ... | White's Hill, Hambrook, near Bristol. |
| | |
| *Dacre, John ... ... ... ... ... ... ... ... | 14 Eaton Crescent, Clifton. |
| *Davies, D. S., M.D. Lond., D.P.H. Cantab. ... | 6 Lansdown Place, Clifton. |
| *Davies, E. D. D. ... ... ... ... ... ... ... | Standish House, Stonehouse, Glos. |
| *Devine, H., *O.B.E.*, M.D. Lond. ... ... ... | Holloway Sanatorium, Virginia Water. |
| *Dix, C. ... ... ... ... ... ... ... ... ... | 11 Victoria Square, Clifton. |
| *Dixon, Helen M., M.B., B.Ch. Brist. ... ... | 10 Whatley Road, Clifton. |
| Dixon, T. B. ... ... ... ... ... ... ... ... | 11 Pembroke Road, Clifton, Bristol. |
| *Drapes, T. L., M.B., B.C., B.A. Cantab. ... | St. Maur, Beaufort Square, Chepstow. |
| *Duerden, J. R., M.B., Ch.B. Brist. ... ... | 149 Bell Hill Road, St. George, Bristol. |
| *Dykes, A. L., M.D. Lond. ... ... ... ... ... | 12 St. Julian Road, Bristol. |
| | |
| *Edgeworth, F. H., M.D., B.C., M.A. Cantab., D.Sc. Lond. ... ... ... ... ... ... ... | 13 Richmond Hill, Clifton. |
| *Eglinton, J. H. C. ... ... ... ... ... ... | Street, Somerset. |
| *Elkington, G. W., M.B., B.S. Lond. ... ... | Bruton, Somerset. |

| | |
|---|---|
| Elliot Bros. Ltd. ... ... ... ... ... ... | *Australian Pharmaceutical Notes & News*, O'Connell Street, Sydney, N.S.W., c/o Grimwade, Ridley & Co., 124 Minories, E.C. |
| Elliott, F. Percy, M.B., C.M. Aberd. ... ... | 113 Grove Rd., Walthamstow, London, E.17. |
| *Elliott, W. H. A., M.B., B.S. Lond. ... ... | 1 Upper Belgrave Road, Clifton. |
| *Every-Clayton, L. E. V., M.D. Lond. | The Quarry, Mullion, Cornwall. |
| | |
| *Faill, C. J. C. ... ... ... ... ... ... ... ... | 19 Portland Square, Bristol. |
| *Fallon, Col. J. ... ... ... ... ... ... ... | 35 Cavendish Road, Henleaze, Bristol. |
| Faraker, E. C., ... ... ... ... ... ... ... | Keinton Mandeville, Somerset |
| Farnfield, W. W.... ... ... .. .. ... ... | Clive Vale, Gillingham, Dorset. |
| *Fawn, G. F. ... ... ... ... ... ... ... ... | 6 Oakfield Road, Clifton. |
| *Fells, A., M.B., C.M. Ed. ... ... ... ... ... | 6 Elton Road, Clifton. |
| *Firth, J. L., M.D., M.S. Lond. ... ... ... ... | 8 Victoria Square, Clifton. |
| *Flemming, A. L., M.B., Ch.B. Brist. ... ... | 48 Pembroke Road, Clifton. |
| *Flemming C. E. S. ... ... ... ... ... ... | Manvers House, Bradford-on-Avon. |
| *Forrester-Brown, Maud, M.D., M.S. ... | 22 Coombe Park, Bath. |
| *Foss, E. V., M.D., Ch.B. Brist. ... ... ... | Summer Hill House, St. George, Bristol. |
| *Fox, F. E., B.A. Camb. ... ... ... ... ... | Brislington House. |
| *Fraser, A. D., M.A., M.B., Ch.B. Glasg. ... | 4 Alexandra Road, Clifton. |
| *Fuller, H. L. ... ... ... ... ... .. ... ... ... | 9 Gay Street, Bath, near Bristol. |
| | |
| *Garden, R. R., M.A., M.B., B.Ch. ... ... ... | 9 Harley Place, Clifton. |
| Garland, E. B., M.B., C.M. Ed. ... ... ... | 114*a* Hampton Road, Redland, Bristol. |
| *Gee, C. A. H., M.B., Ch.B. Brist. ... ... ... | Portbury, Nr. Bristol. |
| *Gibbs, A. N. Godby ... ... ... ... ... ... | 30 Whiteladies Road, Clifton. |
| *Glenny, E. T., M.B., B.S. Lond. ... ... ... | 102 Pembroke Road, Clifton |
| *Golding, Madge E., M.B., Ch.B. Brist. ... | 194 Redland Road, Bristol. |
| *Gordon, R. G. M.D., B.Sc. Ed. ... ... ... | 9 The Circus, Bath. |
| *Gorham, A. P., M.B., Ch.B. ... ... ... ... | 53 Westbury Road, Westbury-on-Trym. |
| *Gornall, W. A., M.B., Ch.B. Brist. ... ... | 23 Windsor Road, St. Andrew's Park, Bristol. |
| *Green, T. A., *D.S.O.*, M.D., C.M. Ed. ... ... | 33*a* Whiteladies Road, Clifton. |
| Griffiths, Cornelius A. ... ... ... ... | 35 Newport Road, Cardiff. |
| *Griffiths, J. S. ... ... ... ... ... ... | Redland Park House, Bristol. |
| *Griffiths, S. J. H., M.B., Ch.B. Brist. ... | Bristol General Hospital. |
| *Groves, E. W. Hey., M.D., M.S. Lond.... ... | 25 Victoria Square, Clifton. |
| | |
| *Hadfield, G., M.D. Lond. ... ... ... ... | Bristol General Hospital. |
| *Hailes Clements, D. G., M.D., C.M. Ed. | 2 Richmond Park Road, Clifton. |
| *Hall, E. George, M.B. Lond. ... ... ... ... | 42 Apsley Road, Clifton, Bristol. |
| *Hall, I. Walker, M.D., Ch.B. Vict. ... ... | Shortwood Lodge, Pucklechurch, Glos. |
| *Ham, C. I., M.B., Ch.B. Brist. ... ... ... ... | Bristol Royal Infirmary. |
| *Harris, H. Elwin, B.A., M.B. Cantab. ... ... | 13 Lansdown Place, Clifton. |
| *Harris, H. E., M.A., M.B., B.Ch. Cantab. ... | 13 Lansdown Place, Clifton. |
| *Harsant, W. H. ... ... ... ... ... ... ... | Tower House, Clifton Down Road Clifton. |
| *Hector, F., M.D. Lond. ... ... ... ... ... | Ham Green Hospital, Pill. |
| *Herapath, C. E. K., *M.C.*, M.D. Lond. ... ... | Ormlie, Clifton. |
| *Hooker, J. A., M.B., Ch.B. ... ... ... ... | 16 St. Edyth's Road, Sea Mills. |
| Hospital, Bristol General (*Secretary*, T. W. Gregg). | |
| *Houghton, L. M., M.B., B.S. Lond. ... ... | 74 Church Road, Redfield, Bristol. |
| Hughes, D., M.B., B.Ch., B.A.O. N.U.I. ... | 12D Cotham Road, Bristol. |
| *Hunter, F. S. ... ... ... ... ... ... ... | c/o Messrs. J. Wright & Sons, Ltd Publishers, Bristol. |

*ILES, A. E., *O.B.E.*, M.B., B.S. Lond. ... ... 17 Victoria Square, Clifton.
INFIRMARY, BRISTOL ROYAL (*Secretary*, E. C. SMITH).
INGLE, C. DURBAN ... ... ... ... ... ... ... Somerton, Somerset.

*JACKMAN, W. A., M.B., Ch.B. Brist.... ... ... 15 Mortimer Road, Clifton.
*JOHNSON, R. G., M.B. Lond. ... ... ... ... 119 Redland Road, Bristol.
*JOLL, C. A., M.B., M.S. Lond. ... ... ... ... 64 Harley Street, London.

*KEMM, N. F., M.B., B.S. Lond. ... ... .. ... 12 Duchess Road, Clifton.
*KENNEDY, W. P., M.D. Dub. ... ... ... ... 3 Gay Street, Bath.
*KERFOOT, STANLEY J. ... ... ... ... ... ... 194 Redland Road, Bristol.
*KINGSTON, S. H., M.B., Ch.B. Brist. ... ... 3 Whiteladies Road, Clifton.
*KYLE, H. G., M.A., M.D. B.Ch. Oxon. ... ... 31 Westbury Road, Bristol.

*LACE, F. ... ... ... ... ... ... ... ... ... ... 5 Gay Street, Bath.
*LEES, E. LEONARD, M.D., C.M. Ed. ... ... ... 22 Richmond Hill, Clifton.
*LEGAT, R. EDDOWES, M.B., C.M. Ed. ... ... Murray House, Staple Hill, Bristol.
LEIGH, W. W. ... ... ... ... ... ... ... ... ... Llansamnor House, Cowbridge, Glam
*LEONARD, R. C., M.D. Durh. ... ... ... ... Bower Ashton, Bristol.
*LESTER, MURIEL A., M.B., B.S. ... ... ... 110 Avon Vale Road, Redfield, Bristol.
*LEVIS, J. S., *M.C.*, M.B., B.Ch., N.U.I. ... 20 Gay Street, Bath.
*LINTON, MARION S., M.B. Lond. ... ... ... 21 Oakfield Road, Clifton.
*LISTER, A. E. J., M.B., B.S. Lond. ... ... 86 Pembroke Road, Clifton.
*LISTER, L. MARGARET ... ... ... ... ... 12 All Saints Road, Clifton.
*LOGAN, H. B. ... ... ... ... ... ... ... ... ... Elmwood, Aberdeen Road, Clifton.
*LUCAS, J. E. ... ... ... ... ... ... ... ... ... 48 Coronation Road, Bristol.
*LUCAS, J. J. S., M.D. Lond. ... ... ... ... ... Highgrove, Wells Road, Totterdown, Bristol.
*LYSAGHT, A. C. ... ... ... ... ... ... ... ... 8 Clifton Hill, Bristol.

*MACDONALD, P. T. T., M.A., M.B., Ch.B. ... 43 Regent Street, Kingswood, Bristol.
*MACFIE, J. D., M.B., Ch.B. ... ... ... ... Winsley Sanatorium, near Bath.
MACKINNON, CHARLES, M.B., C.M., M.A. Glasg. Davaar, Cirencester.
MACLEAN, SIR EWEN J., M.D., C.M. Ed. ... 12 Park Place, Cardiff.
*MACLEOD, DONALD, M.B., C.M. Ed. ... ... 84 Redland Road, Redland, Bristol.
*MACLEOD, G., *M.C.*, M.D., Ch.B. ... ... ... "Wargrave," Clevedon.
*MACWATTERS, J. C., *M.B.E.* ... ... ... ... Almondsbury, near Bristol.
MAGOWAN, B.A., M.B., Ch.B., B.A.O. ... ... Heywood, Pill.
MARLE, S.... ... ... ... ... ... ... ... ... ... Newbridge Road, Bath.
MARSHALL, ARTHUR L., M.B., B.A. Cantab. ... Raveningham, Norwich.
MATHER, J. S., M.B., C.M. Aberd. ... ... ... Lawrence Hill House, Bristol.
*MATHEW, W. E., M.D., C.M. Toronto. ... ... 85 Coldharbour Road, Redland.
*MAYES, F. J. A. ... ... ... ... ... ... ... 79 Pembroke Road, Clifton.
*MCKENZIE, W. G., *M.C.* ... ... ... ... ... 101 Coronation Road, Bristol.
*MCLANNAHAN, J. G. ... ... ... ... ... ... The Mount, Stonehouse.
MIALL, C. L'O. ... ... ... ... ... ... ... Elm Hayes, Paulton, near Bristol.
MIDDLETON-WEST, S.H., *M.C.*, M.B., Ch.B. Vict. ... ... ... ... ... 28 Percival Road, Clifton.
*MILLING, T., M.B., Ch.B. Belfast. ... ... ... 1 Vicarage Road, Southville.
*MOORE, C. A., M.B., M.S. Lond. ... ... ... 56 St. Paul's Road, Clifton.
*MOORE, L. A. ... ... ... ... ... ... ... ... Hillsborough, Cotham Park, Bristol
*MORRIS, A. G. ... ... ... ... ... ... ... ... 18 Westbury Park, Westbury-on-Trym.
MORRIS, L. N., M.B., Ch.B. Brist. ... ... ... 24 All Hallows Road, Upper Easton, Bristol.
*MORTON, C. A., *O.B.E.* ... ... ... ... ... ... 14 Vyvyan Terrace, Clifton.
*MUMFORD, W. G., M.B. Lond. ... ... ... ... 18 The Circus, Bath.
*MUNDY, G S., M.B., B.Ch. Brist. ... ... ... Southmead Hospital, Bristol.
*MURRAY, W. ... ... ... ... ... ... ... ... ... Fairfield, Walton, Clevedon.
*MYLES, G. T. ... ... ... ... ... ... ... ... ... 7 Apsley Road, Clifton.

| | |
|---|---|
| *Neild, Newman, M.B., B.Ch. Vict. ... ... ... | 9 Richmond Hill, Clifton. |
| *Newnham, W. H. C., M.B., M.A. Cantab. ... | 3 Lansdown Place, Victoria Square, Clifton. |
| *Nichols, F. C., *M.C.*, M.B., Ch.B. Brist. ... | 38 Whiteladies Road, Clifton. |
| *Nixon, J. A., *C.M.G.*, M.D. Cantab. ... ... | 7 Lansdown Place, Clifton. |
| *Nott, Winifred, M.B., Ch.B. Brist. ... ... | "Fassiefern," Upper Cranbrook Road, Redland, Bristol. |
| | |
| *Ormerod, H. Lawrence, M.D., B.Ch., R.U.I. | 2 Henleaze Road, Westbury-on-Trym, Bristol. |
| Orr-Ewing, H. J. *M.C.* M.D. Lond. ... ... | English Mission Hospital, Jerusalem. |
| | |
| *Packer, M. E. J., M.B., Ch.B. Brist. ... ... | 90 Filton Road, Filton, Bristol. |
| *Parker, George, M.D., M.A. Cantab. ... ... | 14 Pembroke Road, Clifton. |
| Parkes, J. A., M.B., Ch.B. Liverpool ... ... | 8 South Road, Redland, Bristol. |
| Parkinson, Major G. S., *D.S.O.*, R.A.M.C.... | R.A.M.C. Mess, Grosvenor Place, London, S.W. 1. |
| Parsons, Sir J. H., M.B., B.S., D.Sc. Lond. | 54 Queen Anne Street, London, W. |
| *Perry Bruce, M.B., Ch.B. ... ... ... ... | The General Hospital, Bristol. |
| *Phillips, J. R. P., *C.B.E.* ... ... ... ... ... ... | 34 Archfield Road, Cotham, Bristol. |
| *Phillips, P., M.B., Ch.B. Brist. ... ... ... | Southmead Hospital, Bristol. |
| *Pinkerton, J. M. F.R.C.S. ... ... ... ... | 5 The Circus, Bath. |
| *Pollard, J., M.B., B.Ch., B.A.O. ... ... ... | 52 Cumberland Road, Bristol. |
| Powell, J. J., M.D. Lond. ... ... ... ... ... | 2 Gloucester Road, Bishopston, Bristol. |
| *Pridie, H. H., M.B., C.M. Ed. ... ... ... | 17 Oakfield Road, Clifton. |
| Prowse, D. C., M.B., Ch.B. Brist. ... ... ... | Wigmore House, Thornbury, nr. Bristol. |
| | |
| *Rayner, D. C.... ... ... ... ... ... ... ... ... | 9 Lansdown Place, Clifton, Bristol. |
| *Renton, R. A. S., *M.C.*, M.D. Durh.... ... ... | Emsworth House, Clevedon. |
| Robertson, D., M.B., Ch.B. Ed. ... ... ... | 205 Wells Road, Knowle, Bristol. |
| *Rogers, Beatrice ... ... ... ... ... ... ... | 16 Richmond Hill, Clifton. |
| *Rogers, B. M. H., M.D., B.Ch., B.A. Oxon. ... | 14 Mortimer Road, Clifton. |
| *Rogers, Herbert, M.B., Ch.B. ... ... ... | 11 Clifton Hill, Bristol. |
| *Rolfe, R., B.A., M.B., B.C. Cantab. ... ... | Park House, St. Andrews Road, Avonmouth. |
| *Rowley, A. T.... ... ... ... ... ... ... ... ... | Wrington. |
| *Rutherford, J. M., M.B., C.M. Ed. ... ... | Brislington House, near Bristol. |
| | |
| Salisbury, Walter, M.D., M.S. Lond. ... | Woodlands, 20 Billing Road, Northampton. |
| Savage, W. G., M.D., B.Sc. Lond. ... ... ... | "Bourn," 7 Trewartha Park, Weston-super-Mare. |
| *Scarff, G., M.B., Ch.B., F.R.C.S. Ed. ... ... | Rodney Place, Clifton |
| *Scott, H. H. ... ... ... ... ... ... ... ... ... | 78 Old Market Street, Bristol. |
| *Shaw J. E., M.B., C.M. Ed. ... ... ... ... | 23 Caledonia Place, Clifton. |
| *Shepherd, H. L., M.B., Ch.M. Brist. ... | 29 Victoria Square, Clifton. |
| *Short, A. R., M.D. ... ... ... ... ... ... | 69 Pembroke Road, Clifton. |
| *Short, L. J., M.D., B.S. Lond., M.D. Brist.... | Maywood, Atlantic Road South, Weston-super-Mare. |
| *Smith, G. Cockburn, M.D. Brux. ... ... ... | 12 South Road, Newton Abbot. |
| *Smith, W. Alexander, M.B., M.A. Oxon. ... | 70 Pembroke Road, Clifton. |
| *Smyth, Richard, M.A., M.D. Dub. ... ... | Burnley, Westbury-on-Trym, Bristol. |
| *Smythe, H. J. D., *M.C.*, M.B., M.S. ... ... | 15 Eaton Crescent, Clifton. |
| *Somers, Mary, Miss, L.S.A. ... .. ... ... | 2 Ashcombe Road, Weston-super-Mare. |
| *Spoor, A., M.B., B.Ch. Cantab.... ... ... ... | The Hydro, Bristol. |
| *Staniland, M. F. ... ... ... ... ... ... ... | 255 Stapleton Road, Bristol. |

| | |
|---|---|
| *STATHAM, R. S. S., *O.B.E.*, M.D., M.Ch. Brist. | 10 Beaufort Road, Clifton, Bristol. |
| *STEWART, A. L., D.M., D.Ch. Brux. ... ... | 31 Howard Road, Southville, Bristol. |
| *STOCK, W. S. V., M.B. Lond. ... ... ... | 1 Mortimer Road, Clifton. |
| *STROVER, H. W. M., *O.B.E.*, M.B., Ch.B. Aberd. ... ... ... ... ... ... ... | 24 Gloucester Road, Bishopston, Bristol. |
| *SYMES, J. O., M.D. Lond. ... ... ... ... | 71 Pembroke Road, Clifton. |
| *TASKER, D. G. C., M.B., M.S. Lond. ... ... | 16 All Saints Road, Clifton. |
| TAYLOR, A. L. ... ... ... ... ... ... | The Royal Infirmary, Bristol. |
| *TAYLOR, H. N., M.A. Cantab., M.D. Dub.... | Hillside, Ebbw Vale. |
| *TEMPLE, G. H., M.B., C.M. Ed. ... ... ... | Ailanthus, Weston-super-Mare. |
| *TERRY, C. H., M.A., M.B., B.Ch. Oxon. ... | 15 The Circus, Bath. |
| THIN, JAMES ... ... ... ... ... ... ... | 54 & 55 South Bridge, Edinburgh. |
| THOMPSON, J., M.B., Ch.B. ... ... ... ... | Eastbourne House, Devizes. |
| *THOMAS, J. D., M.B. Cantab. ... ... ... | Northwoods, Winterbourne. |
| *TODD, A. T., *O.B.E.*, M.B., Ch.B. Ed. ... | Clayton, Clifton Park, Clifton. |
| *TRIST, J. R. R., *M.C.* ... ... ... ... ... | 120 Henleaze Road, Clifton. |
| *TRYON, V., M.B., Ch.B. Brist. ... ... ... | 3 Harley Place, Clifton. |
| *VICKERY, W. H. ... ... ... ... ... ... | 29 Trewartha Park, Weston-super-Mare |
| *VINCENT, A., M.B., Ch.B. ... ... ... ... ... | 37 Chesterfield Road, St. Andrews Park. |
| *WALKER, CYRIL H., M.B., B.A. Cantab. | 8 Oakfield Road, Clifton. |
| *WALLACE, JOHN, *O.B.E.*, M.B., C.M. Ed. | 10 Knightstone Road, Weston-super-Mare. |
| *WALTERS, C. F. ... ... ... ... ... ... | 5 Mortimer Road, Clifton. |
| *WANKLYN-JAMES, C. W., *O.B.E.* ... ... | 3 Mortimer Road, Clifton. |
| *WARREN, R., M.A., M.D. Oxon. ... ... ... | Ellenborough Hall, Weston super-Mare. |
| *WATERHOUSE, R., M.D.Lond. ... ... ... | 25 The Circus, Bath. |
| *WATSON-WILLIAMS, E., *M.C.*, B.A. Cantab. | 12 Victoria Square, Clifton. |
| *WESTON, B. A. A., M.B., B.Ch. Brist. ... | 30 Cotham Grove, Bristol. |
| *WEATHERHEAD, E., M.B., B.Ch. Cantab. ... | 115 Queen's Road, Clifton, Bristol. |
| WESTON, B. A., M.B. ... ... ... ... ... ... | Brookside, Admaston, Wellington, Shropshire. |
| *WHICHER, A. H. ... ... ... ... ... ... | 115 Queen's Road, Clifton. |
| *WHITE, E. B. C. ... ... ... ... ... ... | Mental Hospital, Fishponds, Bristol. |
| WHITEHOUSE, H. BECKWITH, M.S. Lond. ... | 52 Newhall Street, Birmingham. |
| WIGAN, C. A., M.D. Durh., J.P. ... ... ... | Deepdene, Portishead. |
| WIGMORE, J., M.D., R. U. I. ... ... ... | 16 Queen Square, Bath. |
| WILLCOX, R. LEWIS ... ... ... ... ... | 97 London Road, Salisbury. |
| WILLIAMS, S. R., M.B. Lond. ... ... ... | Buckfastleigh, Devon. |
| *WILLS, W. K., *O.B.E.*, M.B., B.C. Cantab. | 19 Whiteladies Road, Clifton. |
| *WOOD, DUNCAN ... ... ... ... ... ... | 5 Eaton Crescent, Clifton. |
| *WOOD, W. V., *M.C.*, B.A. Cantab. ... ... | Henley Lodge, Yatton, Som. |
| *WRIGHT, A. J., M.B., B.S. Lond. ... ... | 2 Clifton Park, Clifton. |
| *ZEALAND, L., M.D. Man. ... ... ... ... | Brecon Lodge, Westbury-on-Trym, Bristol. |

### Publications Received.

From Wm. Heinemann (Medical Books) Ltd.:

*Diseases of the Nose, Throat and Ear.* By W. S. Syme, M.D., F.R.F.P. & S.G., F.R.S.E. 2nd Edition. 1927. Price 12s. 6d. net.

*Practical Birth Control.* By Ettie A. Hornibrook. 1927. Price 3s. 6d. net.

From Kegan, Paul, Trench, Trubner & Co.:

*Medicine: and the Man.* By M. Culpin, M.D., F.R.C.S. 1927. Price 2s. 6d. net.

*The Constitutional Factor in Disease.* By A. F. Hurst, M.D., F.R.C.P. 1927. Price 2s. 6d. net.

From H. K. Lewis & Co., London:

*A Textbook of Infectious Diseases.* By E. W. Goodall, O.B.E., M.D., B.S. (Lond.). 3rd Edition. 1928. 30s. net.

*Treatment by Manipulation.* By A. G. T. Fisher, M.C., F.R.C.S. (Eng.). 2nd Edition. 1928. 9s. net.

From E. & S. Livingstone, Edinburgh:

*Dental Surgery.* By J. L. D. Buxton, L.D.S., R.C.S., L.M.S.S.A. (*Outlines of Dental Science,* Vol. VIII.) 1927. Price 7s. 6d. net.

*Dental Pathology.* By J. L. D. Buxton, L.D.S., R.C.S., L.M.S.S.A. (*Outlines of Dental Science,* Vol. VII.) 1927. Price 7s. 6d. net.

*The Examination of the Central Nervous System.* By D. Core, M.D. (Manc.), F.R.C.P. (Lond.). 1928. 8s. 6d. net.

*Advice to the Expectant Mother on the Care of her Health.* By F. J. Browne, M.D., D.Sc., F.R.C.S.E. 2nd Edition. 1928. Price 6d. net.

From Humphrey Milford, O.U.P.:

*Post-mortem Appearances.* By Joan M. Ross, M.D., B.S. (Lond.), M.R.C.S., L.R.C.P. With a Preface by E. H. Kettle, M.D. 2nd Edition. 1928. Price 7s. 6d. net.

*The Abdominal Surgery of Children.* By L. E. Barrington-Ward, Ch.M., F.R.C.S. (Edin.), F.R.C.S. (Eng.). 1928. Price 15s. net.

*The Surgical Treatment of Malignant Disease.* By Sir H. J. Waring, M.S., M.B., B.Sc. (Lond.), F.R.C.S. 1928. Price 50s. net.

From John Wright & Sons Ltd.:

*British Journal of Surgery.* January, 1928. 12s. 6d. net. Annual Subscription 42s.

---

### Bristol Medico-Chirurgical Journal.

### NOTICE TO CONTRIBUTORS.

Authors of original articles are entitled to receive twenty-five reprints, without covers, free of charge. Additional reprints with or without covers may be obtained at the following rates (provided notice is given at the time of returning proofs):—

**ADDITIONAL COPIES.**

| | Copies | 8 pages | 16 pages |
|---|---|---|---|
| Without cover | 25 copies, | 4/- | 8/- |
| | 50 ,, | 8/- | 16/- |
| With plain cover (no name or title) | 25 ,, | 9/- | 13/- |
| | 50 ,, | 15/- | 23/- |
| With special cover (i.e. Author's name and title of paper) | 25 ,, | 12/- | 16/- |
| | 50 ,, | 18/- | 26/- |

**Illustrations.**—For 25 copies of one page, **9d.** extra. For 50 copies of one page, **1/6** extra.

Note.—Contributors requiring Covers on their free Reprints may obtain same at the following rates:—

| | 25 copies | 50 copies |
|---|---|---|
| Plain Cover | 5/- | 7/- |
| Cover with Author's name and title of article | 8/- | 10/- |

*Second Edition. Fully Revised and Enlarged. Demy 8vo, 464 pp. 234 Text Illustrations and 12 Plates (of which 8 are in Colour).* **20s.** *net, Postage* **9d.**

### DISEASES OF THE NOSE, THROAT AND EAR

**For Practitioners and Students.**

**Edited by A. LOGAN TURNER, M.D., LL.D., F.R.C.S.E.,**

*Consulting Surgeon, Ear and Throat Department, Royal Infirmary, Edinburgh.*

With the collaboration of J. S. FRASER, M.B., F.R.C.S.E., W. T. GARDINER, M.C., M.B., F.R.C.S.E., J. D. LITHGOW, M.B., F.R.C.S.E., G. EWART MARTIN, M.B., F.R.C.S.E., and DOUGLAS GUTHRIE, M.D., F.R.C.S.E.

"May confidently be recommended for the use of students and practitioners."—*Lancet.*

"Will prove of the greatest value."—*Journal of Laryngology and Otology.*

---

*With 71 Fully Coloured Cystoscopic Appearances in 12 Plates, many Pyelograms and other Illustrations and Diagrams. Large 8vo. 272 pp.* **25s.** *net. Postage* **9d.**

### CYSTOSCOPY.

**A Theoretical and Practical Handbook, containing Chapters on Separate Renal Function and Pyelography.**

**By JAS. B. MACALPINE, F.R.C.S. (Eng.),**

*Hon. Surgeon and Surgeon-in-Charge of the Genito-Urinary Dept., Salford Royal Hospital Manchester.*

"This book is a remarkable achievement, we consider it to be one of the best monographs we have ever read, and we predict that it will rapidly become the standard work on the subject."—*The British Journal of Surgery.*

---

*Recently Published. Large 8vo. 217 pp. With 261 Original Illustrations some of which are in Colour.* **21s.** *net. Postage* **6d.**

### PHYSICAL SIGNS IN CLINICAL SURGERY

**By HAMILTON BAILEY, F.R.C.S. (Eng.),**

*Surgeon, Dudley Road Hospital, Birmingham.*

"The author is to be congratulated upon the success with which he has carried out his task. A feature of the book is the abundance of illustrations, all of which, in addition to the excellence of reproduction, are informative."—*Lancet.*

"This book marks an epoch in books devoted to clinical signs. Nowhere before have we been able to find such extreme clarity of photography aiding such a scholarly exposition of this subject."—*St. Thomas's Hospital Gazette.*

---

*Latest English Impression. With 1,086 Illustrations in the Text, and 20 full-page Plates. Super Royal 8vo, 824 pp. Cloth gilt.* **63s.** *net.*

### URGENT SURGERY.

**By FELIX LEJARS.**

**Translated from the Eighth French Edition by W. S. DICKIE, O.B.E., F.R.C.S., and ERNEST WARD, M.A., M.D., F.R.C.S.**

"Will prove of the greatest possible help to any medical man not specially versed in surgery who is called upon to perform a major operation, and even specialist surgeons will derive many useful hints from its perusal."—*British Journal of Surgery.*

---

*Ninth Edition. Fully Revised. With 343 Text Illustrations and 11 Plates.* **21s.** *net.*

### PYE'S SURGICAL HANDICRAFT.

**A Manual of Surgical Manipulations, Minor Surgery, and other matters connected with the work of House Surgeons, Surgical Dressers, etc.**

Edited and largely re-written

**By W. H. CLAYTON-GREENE, C.B.E., B.A., M.B., B.Ch. (Camb.), F.R.C.S.**

*Late Consulting Surgeon to St. Mary's Hosp., and Lecturer on Surgery in the Medical School, etc.*

With special Chapters by distinguished contributors.

"Full of information invaluable to the student, the house surgeon, and the general practitioner."
*Lancet.*

***Illustrated Catalogue free on application.***

---

**BRISTOL, ENG. JOHN WRIGHT & SONS LTD.**

**LONDON: SIMPKIN, MARSHALL, HAMILTON, KENT & CO. LTD.**

